# Are SAARC countries prepared to combat COVID-19 to save young, working-age population?

**DOI:** 10.3934/publichealth.2020036

**Published:** 2020-07-01

**Authors:** Farhana Sultana, Hasan Mahmud Reza

**Affiliations:** 1 Department of Political Science and Sociology, North South University, Bashundhara R/A, Dhaka 1229, Bangladesh; 2 Department of Pharmaceutical Sciences, North South University, Bashundhara R/A, Dhaka 1229, Bangladesh

**Keywords:** COVID-19, SAARC, young population, climate

## Abstract

The COVID-19 outbreak has expanded across the globe. Most of the countries are launching different measures to stop the transmission of this virus. However, the death toll is steadily rising. Strikingly the rate of coronavirus infection among the young-age population is the highest in SAARC countries as more than 80% population of the SAARC countries are young who constitute the working-age group. The disease transmission also occurs at a slower rate presumably due to diverse lifestyles of different ethnicities, immunity and genetic traits; but not because of the hot and humid weather despite previous assumptions. Since SAARC countries comprise 23.75% of the world population and the largest portion of these people is the young working-class, some immediate measures need to be implemented to save these valuable lives from COVID-19. Till now, there is no specific treatment or vaccine available; hence timely-taken preventive measures are the only hope that can save the people of this region. Here we have demonstrated an altered disease transmission pattern in people of SAARC countries, measures initiated by the governments, causes of failure and further actions to be taken to control disease transmission.

## 1. Introduction

COVID-19 has halted people's regular lives, and now holds the world in its clutches. The deadly novel Coronavirus (2019-nCoV2 or SARS-CoV-2), first detected in Wuhan in December 2019, spread rapidly around the world [1]. Based on observed clinical manifestations caused by this virus, the ailment had initially been named “novel coronavirus-infected pneumonia” (NCIP) before it was renamed “Corona Virus Disease 2019” (COVID-19) [1],[2]. COVID-19 is an illness characterized by severe respiratory distress, and it is more dangerous than the flu virus. WHO demonstrates that 2019-nCov is highly contagious, with each infected person being able to pass it to 1.4∼2.5 people [3]. Considering the enormous potential of this virus to spread and infect people, WHO declared COVID-19 a global pandemic on March 11, 2020 [4]. At present, COVID-19 transmission has occurred in 213 countries and territories. More than 8.9 million people are infected and 0.46 million have already died [5]. The death toll is rising, so far unrestrained. The SAARC countries, home to 23.75% of the world population and comprising of eight nations—Afghanistan, Bangladesh, Bhutan, India, Maldives, Nepal, Pakistan and Sri Lanka—have not yet experienced the devastating ramifications of COVID-19 [6],[7]. Studies demonstrate that the viral transmission and propagation primarily depends on viral intrinsic factors and local factors including environmental conditions and host status in terms of immunity, lifestyle, ethnicity, etc [8]. The socio-economic conditions of the majority of people in this region are not conducive to maintain certain hygienic standards to prevent infection transmission [9]. As the transmission of SARS-CoV-2 follows a slow dynamic in this region, and a huge number of young population is infected, we aimed to explain the possible determining factors behind it. Further, we expanded the discussion focusing on the overall preparedness and actionable strategies to prevent further spreading of COVID-19.

## 2. Methodology

### 2.1. Study design

In this population-based observational study, we used reports from government websites (iedcr.gov.bd, covid.gov.pk, corona.mygov.in, covid19.gov.lk, https://heoc.mohp.gov.np/, update-on-novel-corona-virus-covid-19/, moh.gov.bt/novel-coronavirus-2019-ncov/, https://covid19.health.gov.mv/dashboard/, af.usembassy.gov/covid-19-information/), official WHO site (https://covid19.who.int/), worldometer, news media and televisions. These are publicly available data reported directly by public health authorities or by state media and are linked to an online source, which can be accessed for more detailed information.

### 2.2. Data compilation

We closely monitored updates on worldometer and other sources used in this study between March 01, 2020, and June 21, 2020, to extract relevant information on subjects in near real-time, and reports of daily case counts.

## 3. Results and discussion

### 3.1. COVID-19 is not age-specific

COVID-19 shows a different transmission pattern in people of SAARC countries. Among 753,213 confirmed cases from the three most populous countries—India, Pakistan and Bangladesh, it has been observed that a surprisingly higher percentage of the infected patients comprise of young people aged 20–39 years: 44.10% in India, 45.0% in Pakistan and 67.7% in Bangladesh. While the “most high-risk” age group (≥ 60 years) as defined by China and Europe is less infected in these countries, a higher infection rate in population aged 0–19 has been observed [10],[11]. To understand the underlying reasons for the increased number of COVID-19 cases in the young population in these countries, it is worth mentioning that on average, 85% of the population in India, Pakistan and Bangladesh is younger than 50 years [12],[13]. Moreover, the 20–39 age group comprises most of the working-class; therefore, they are required to venture outside in spite of the risk of infecting others or becoming exposed to infected people. A large portion of this age group does not have a stable monthly income and rather live on daily-basis jobs. As a result, they are compelled to break containment and consequently ignore social distancing protocols, which impacts to further increase the infection rate. These findings suggest that COVID-19 is not age-specific and all age groups are susceptible to infection. A similar infection trend in the USA has also been reported [14].

### 3.2. Garments workers appear as a potential vector to increase further infection among the young population

Over 45 million people in India, 4 million in Bangladesh and 2 million in Pakistan are garment workers. This large work-force is compelled to go to factories as there is no work-from-home option for them. Consequently, convincing people to stay indoors or to maintain a social distance or lockdown is difficult, even if it is to protect them from a deadly virus. Again 95% of the garment workers in India, 80% in Bangladesh and 75% in Pakistan are female workers who carry additional responsibilities to take care of their families and thus come in close contact with other members of the family, which poses further risk to infect whole families.

Many of the workers do not have written job contracts and there is a practice of improper hiring and firing of employees in these garment factories. Proper labor laws are not followed in many factories although governments try to enforce such laws to protect millions of garment workers from labor rights abuses. As a result, garment workers always remain in fear of losing jobs and therefore, they try to comply even with the unlawful instructions that are imposed on them by the factory management. In the background of the COVID-19 crisis, these poor people are further afraid that the outbreak will personally impact them financially. A very recent decision of bringing the garment workers back to the factories by relaxing lockdown has impacted a lot in increasing the infection rate in Bangladesh. More than a thousand garment factories in the suburbs of the capital, Dhaka, have begun functioning. So the millions of people rush to factories in Dhaka and nearby cities from their homes in villages on foot or by crowded vehicles. These crowds are now potential vectors of the COVID-19 pandemic in Bangladesh. More factories are likely to open soon. If this class of young, working-age population is not made educated with the minimum health literacy to guard themselves while working in the factories, a horrendous situation is likely to occur.

### 3.3. SARS-Cov-2 transmission is not seasonal or climate-dependent

Initially, it was hypothesized that COVID-19 transmission may be restricted by a hot and humid climate. As the SAARC countries are in the tropical region with a hot and humid climate, such a hypothesis caused these countries to show a delayed response in preparedness, which in turn, has made the situation horrific. The three most populous countries in this region, India, Pakistan and Bangladesh, are now experiencing a high rate of infection and death toll. The transmission is increasing at an alarming rate. These observations suggest that COVID-19 transmission does not depend on raised temperatures, which is in agreement with recent reports demonstrating that case counts of COVID-19 did not decline when the weather became warmer [15],[16]. Nevertheless, COVID-19 transmission is reasonably slower in most of South Asian countries compared to European, African and North American countries, which may be explained by a different lifestyle, ethnicity, genetic traits and preexisting partial immunity of the people of these countries. Further research will be required to establish this notion.

### 3.4. Aberrant responses to COVID-19 by SAARC countries

Having left China devastated, 2019-nCov hit European countries, notably Italy, Spain, France, UK and Germany, more catastrophically [17]. At present, the United States has the highest death rate. The first cases of COVID-19 were detected much later in South Asia compared to China and Europe. Initially, the increase of positive cases had been slow; however, the situation has since gained momentum and the infection rate is now rising at an exponential rate. The three most affected SAARC countries are India, Pakistan and Bangladesh (Table 1). It is evident that the death toll is highest in India, followed by Pakistan and Bangladesh. It is worth noting that among these three countries, India has performed the highest number of COVID-19 tests per million people, followed by Pakistan and Bangladesh (Table 1). However, the ratio of the number of COVID-19 tests to the total population is low for these countries,therefore, there is a strong possibility that the actual number of COVID-19 patients and deaths is higher in all three countries since many deaths in periphery towns and villages as reported in the news media are not being identified due to lack of proper management.

We further observed that the mortality rate among infected cases is the highest in Bangladesh among all SAARC countries. One reason may be that Bangladesh had been quite tardy in initiating the necessary measures against COVID-19 transmission. In fact, many people with clear symptoms of COVID-19 were left untested initially because of a lack of test kits. Those who arrived from the affected countries were neither properly screened at airports nor quarantined with proper monitoring. Therefore, they dwelt normally with other people and infected many locals unknowingly. The scenario in other SAARC countries was not much different. These countries showed a significant lack of preparedness despite having had sufficient time as the disease emerged much later in this region. These countries had most likely turned a blind eye to the devastating nature of the virus and instead had opted to downplay the global phenomenon to calm and appease their citizens. The people believed the unproven idea that a warm climate would protect them against COVID-19; the lack of urgency among citizens and the absence of a prior management plan by the government thus allowed free transmission of this virus. India and Pakistan showed a similar delayed response. As a consequence, only an insignificant portion of the population has been tested in Bangladesh, India, Pakistan and Afghanistan as evidenced by the worldometer data (Table 1). By contrast, Sri-Lanka, Maldives, Nepal and Bhutan realized the annihilating power of the COVID-19 crisis early enough and acted accordingly by launching measures such as closing borders and schools, implementing curfews, quarantine and/or lockdowns on districts, enforcing social distancing, etc, and were able to limit the transmission effectively. However, people traveling to the Maldives from some affected areas during the past couple of weeks have catalyzed a gradual change in this scenario; only a few deaths have been reported in Maldives, Sri Lanka and Nepal while none in Bhutan till now [5].

**Table 1.** Status of COVID-19 in SAARC countries*.

Sl. No.	Country	No. of Infected Cases	No. of Deaths	Tests/Million Population
1	Afghanistan	28,833	581	1,670
2	Bangladesh	112,306	1,464	3,736
3	Bhutan	68	-	29,758
4	India	422,526	13,509	4,934
5	Maldives	2,187	8	65,775
6	Nepal	9,026	23	15,019
7	Pakistan	176,617	3,501	4,855
8	Sri Lanka	1,950	11	4,441

Note: * Worldometer, https://www.worldometers.info/coronavirus/, accessed June 21, 2020.

### 3.5. Physicians and nurses are at severe risk of infection

Physicians and health workers are at the forefront of the war against COVID-19. They need proper PPEs as recommended by WHO to guard themselves first. In the meantime, a large number of physicians and nurses have been detected with COVID-19; meanwhile, fifty physicians have died only in Bangladesh as they were not provided with quality PPE and the overall hospital management was horrible. This has further intensified the crisis of healthcare professionals. In all SAARC countries, the number of physicians and nurses is inadequate compared to the national need. If the death trend of the frontliners continues in such a way, there will be a severe shortage of physicians which poses a big threat to the treatment of COVID-19 patients. The infection rate is still increasing within this group of people. Several hospitals have been locked down. If these countries fail to keep healthcare professionals healthy, the disaster will be dreadful. The COVID-19 infection has also occurred in people from Media, Law-enforcement and Cleaning departments who are in the battlefield to perform their jobs even in this crisis hour. These groups are also required to use PPE as recommended by WHO guidelines for safety.

### 3.6. Measures taken in SAARC countries

Most SAARC countries, by this time, are trying to impose regulations but not in an effective way. Bangladesh has recently introduced some measures like social distancing, regional lockdown, closing borders and closing educational institutions. In Bangladesh, India, Sri Lanka and Pakistan, policing has been strengthened by deploying Army and Navy in the streets. However, because of pressure from different sectors like mills and factories, small traders, hawkers, daily labors, the governments of SAARC countries are behaving lenient and may not continue the lockdown, which will be detrimental.

### 3.7. Attitude, knowledge, poverty and inadequate resources accelerate disease transmission in SAARC countries

SAARC countries have the largest number of poor people in the world. About 33.4% population in this region is living on less than US$ 1.90/day income [18]. This large population needs to go outside for day-to-day livelihood, which further aggravates the situation as this allows more human-to-human transmission resulting in an exponential increase in case numbers as observed in China [19]. This group of people is underprivileged, less educated, fundamentally ignorant in health knowledge and careless in regards to government directives essential for their safety. A huge number of population living in both rural and urban areas of these countries are emotionally driven: they believe various superstitions and depend on luck rather than consciously working to change the situation. These people gather in mosques, temples and churches to perform their religious activities and infringe social distancing protocols. They also continue to organize social gatherings without acknowledging the health regulations at this time of crisis.

The health-care system in most of these countries is not developed enough to tackle any disaster like COVID-19. Hospitals and health-care centers are not equipped with the minimum requirements including central oxygen supplies, ventilators and ICUs that are essential for COVID-19 patients. Personal Protection Equipment (PPE) for classified people is a critical issue in these countries. At the beginning, there were no such PPEs; recently the supply of PPE has been increased but the quality has not been maintained, which has made the situation more terrible. More than a thousand physicians and health workers in India, Pakistan and Bangladesh have been infected by COVID-19 because of wearing poor quality PPEs. In the case of Afghanistan, the health sector is severely disrupted compared to other SAARC countries. War-torn Afghanistan is not capable of combatting COVID-19 with its available resources. As per WHO reports, there is a single isolation center with a capacity of 100 beds and one diagnostic lab capable of testing only fifty samples per day [20]. Most people are illiterate with poor health knowledge. Hence, the COVID-19 situation is likely to worsen in Afghanistan if international organizations do not extend their medical and financial aid sufficiently.

### 3.8. Lack of integrated plans and implementation is a barrier in the proper management of COVID-19 in SAARC countries

So far many initiatives have been put into action in Afghanistan, Bangladesh, Pakistan and India very recently to prevent COVID-19 transmission. However, the plans have not been developed through an integrated process combining experts from the relevant areas. There is a huge antagonism between ruling and opposition parties. Private sectors are also quite reluctant to move forward with the government. Different ministries within the government including Health Ministry, Disaster Management and Relief Ministry, Ministry of Home Affairs and Ministry of Defence are working together, albeit a lack of coordination is often reported. Local organizations like the National Centre for Disease Control Institute of India (NCDC), Pakistani National Institute of Health (NIH), Institute of Epidemiology, Disease Control and Research of Bangladesh (IDECR), Epidemiology and Disease Control Division of Nepal (EDCD), Royal Centre for Disease Control of Bhutan (RCDC) and the Epidemiology Unit of Sri Lanka are working closely with the governments in updating the countries' COVID-19 statuses, creating awareness and developing interim action plans in accordance with the WHO guidelines. But again the implementation and monitoring of these measures are negligent. A lack of coordination among different stakeholders is visible. The corruption in the administration is another important factor that worsens the situation in these countries. Only an insignificant number of NGOs, research centers, medical colleges and universities are also providing voluntary supports in various ways to combat COVID-19 in SAARC countries.

### 3.9. Actions to be taken urgently and strictly

The World Bank has warned that South Asia may experience its worst economic performance because of COVID-19 pandemic. Despite this fact, the protection of public health should be given the highest priority. SAARC countries are densely populated and the majority of people can neither afford nor care to maintain a minimum level of hygiene. A pandemic like COVID-19 can snatch many thousands of lives overnight. Considering the current pattern of COVID-19 transmission, it is predicted that the sharp peaks of COVID-19 infections and deaths observed in other countries may not be mimicked in SAARC countries, rather a sustained plateau graph is anticipated, which suggests that all preventive measures need to be continued for a longer time. Therefore, the leaders of SAARC countries need to take the following actions immediately to avoid a massive death toll like those observed in Europe and the USA:

1. Identify clusters of COVID-19 cases and enforce lockdown with strict monitoring.

2. Redesign training programs and communication activities for more effective dissemination of information related to the COVID-19 epidemic. Television, online newspaper and social media can be used effectively with celebrities' participation to educate and convince people to comply with health rules [21]–[23].

3. Make quarantine centers in each factory as well as locality to prevent any possibility of the diffusion of COVID-19 to other people who are dwelling in proximity. Organizational measures including workplace hygiene, tracking the health status of employees, avoidance of large gatherings and maintenance of interpersonal distance must be in effect [24],[25].

4. Aware people to remain in-doors, use face-mask and maintain social distancing. These are “must-do” strategies that help to prevent the spreading of coronavirus via human-to-human transmission most effectively. Also make them aware of how to dispose of face-masks, gloves and PPEs following the decontamination process as suggested by the WHO guidelines. This is essentially important to minimize the environment-to-human transmission of the virus [9],[22].

5. Increase aid mainly foods, medicines, disinfectants/soap and essential commodities to the poor to keep them in-doors. Governments need to put efforts to ensure financial security and optimal mental health and avoid psychological reflexes among general public [22].

6. Provide PPE to health professionals and increase the supply of disinfectants sufficiently as physicians and allied health professionals are the frontliners who need to survive to win this battle. The total hospital management system including triage, regular sanitization, proper medical waste disposal, rationing manpower, etc. need to be maintained properly to prevent infection diffusion.

7. Increase both antigen and molecular testing for effective containment.

8. Increase treatment facilities including temporary hospitals, dedicated COVID-19 hospitals, continuous oxygen supply, high flow nasal cannula, ventilators, ICUs and medicines for an anticipated number of patients. ICU doctors and nurses also need to be increased (Sterpetti).

9. Allocate special assistance and support for Afghanistan.

SAARC can play an effective role by aiding cooperation among the member countries and thus, save the large working-age population from COVID-19. It is worth mentioning that the member countries have agreed to raise a common SAARC Corona Emergency Fund to secure the people of this region. One limitation of the study is that we used data from government sites that may not always reflect the actual scenario. Another limitation was that the study could not include sufficient real-time data in the context of Afghanistan due to its unavailability on the web.

## 4. Conclusion

COVID-19 is a big threat to mankind. SAARC people will experience a huge death toll if effective and aggressive measures are not taken immediately. Increasing awareness and implementing timely measures can minimize this loss to a large extent. Therefore, both leaders and the general public need to act responsively.
